# Action of melatonin on squamous cell carcinoma and other tumors of the oral cavity (Review)

**DOI:** 10.3892/ol.2014.1813

**Published:** 2014-01-20

**Authors:** ANTONIO CUTANDO, ANTONIO LÓPEZ-VALVERDE, JOAQUIN DE VICENTE, JULIAN LÓPEZ GIMENEZ, ISAAC ALIAS CARCÍA, RAFAEL GOMEZ DE DIEGO

**Affiliations:** 1 Department of Special Care in Dentistry, School of Dentistry, University of Granada, Granada 18071, Spain; 2 Department of Surgery, School of Dentistry, Faculty of Medicine, University of Salamanca, Salamanca 37007, Spain; 3 Department of Surgery, School of Dentistry, University Alfonso X, Madrid 28691, Spain

**Keywords:** melatonin, carcinoma, tumors

## Abstract

Melatonin (MLT; *N*-acetyl-5-metoxy-tryptamine) is a hormone that is principally synthesized in the pineal gland. MLT has been shown to exhibit a variety of functions. The hormone, which is a free radical scavenger, plays an immunomodulatory role, stimulates the proliferation and synthesis of type I collagen and promotes bone formation. Moreover, MLT exerts oncostatic activity through several biological mechanisms, including antiproliferative functions, stimulation of anticancer immunity, modulation of oncogene expression and anti-inflammatory, antioxidant and antiangiogenic effects. In addition, MLT inhibits human cancer cell growth in culture, and previous clinical studies have also confirmed its anticancer properties *in vivo*. With regard to the underlying mechanisms of MLT in tumor processes, including oral cavity tumors such as epidermoid carcinoma, knowledge of the role played by the MT1 and 2 membrane receptors, MT3 and the calmodulin cytosolic binding sites, as well as the nuclear receptors of the RZR/ROR family, is increasing. It has been hypothesized that exogenous restoration of MT1 (MTNR1A) expression inhibits the growth of oral squamous cell carcinoma cells lacking the expression of the receptor. The tumor suppressing functions of MLT and the presence of the MT1 receptor in various tumors indicate that the receptor may play a pivotal role in oral carcinogenesis. The current review discusses the clinical significance of MLT in oral cancer.

## 1. Introduction

Melatonin (MLT) or *N*-acetyl-5-metoxy-tryptamine, is a hormone synthesized in various organs of the body, but principally in the pineal gland (1). The pineal gland produces MLT in a circadian manner, synchronizing a number of biological processes in a 24-h, day-night rhythm. An internal 24-h time keeping system (biological clock) regulated by MLT controls the sleep-wake cycle (2). MLT affects numerous aspects of circadian and circannual rhythms, including sleep, actions that are mediated by the binding of indoleamine to membrane receptors (3,4). A number of studies have shown the various actions of MLT with regard to aspects of intracellular functions associated with mechanisms that are independent of the action of the hormone on the cellular membrane receptors. For these actions, nuclear receptors for MLT have been identified in the cells of the central nervous system and peripheral organs (5–7). Since MLT has lipophilic properties, the hormone is able to reach intracellular organelles, including the nucleus and mitochondria (8). In the cell interior, MLT is capable of binding to specific cytosolic proteins, including protein kinase C (9,10), calmodulin (10,11), calreticulin (12) and quinone reductase-2 (QR2) (13).

Currently, MLT is not regarded as a hormone in the classical sense, but functions as a cell protector since it is not synthesized in a single organ and does not exert effects upon a specific target organ. Studies have shown that MLT is a molecule with paracrine, autocrine and antioxidant effects that exerts diverse receptor-dependent and -independent actions (14,15). Overall, MLT exhibits homeostatic functions and pleiotropic effects relevant to cell protection and survival mechanisms (16).

MLT is produced in several organs, and MLT-forming enzymes are found in a number of tissues, including the retina (17,18), ovaries (19,20), gastrointestinal tract (21,22) and cells of the immune system (23). MLT is released into the blood and then passively diffuses into the oral cavity via saliva and the oral mucosa (24). MLT in the saliva is found in concentrations up to 70% lower than those in blood, and considering that MLT is bound to plasma proteins, salivary MLT represents the percentage of free MLT that is not albumin-bound (25). The measurement of salivary MLT is a useful non-invasive technique for monitoring MLT circadian rhythmicity (26). The major metabolite of MLT is 6-hydroxymelatonin sulfate (derived from 6-hydroxylation and conjugation primarily to sulfates in the liver), which is excreted in the urine in a larger amount at night than during the day (1). MLT is also converted to cyclic 3-hydroxymelatonin, presumably in all cells, and this metabolite is excreted in urine (27).

## 2. Oncostatic properties of MLT

It has been shown that MLT exhibits oncostatic properties on a wide variety of tumors, including prostate, colorectal, neural, ovarian, breast and cervical cancers, and sarcomas, hepatocarcinomas, melanomas, larynx carcinomas and skin carcinomas (28). The oncostatic effects of MLT have been well studied in hormone-dependent tumors. Extensive evidence on the oncostatic activity of MLT is based on *in vitro* studies carried out in cell lines derived from human tumors and murine tumoral models. The general conclusions of these studies are that MLT inhibits cell proliferation and induces apoptosis in the majority of tumor cell lines. Mechanisms of cancer inhibition by MLT include antioxidant effects, the regulation of estrogen receptor expression and transactivation, modulation of the enzymes involved in the local synthesis of estrogens, modulation of the cell cycle, differentiation and apoptosis, inhibition of telomerase activity, antiangiogenesis, prevention of circadian disruption, activation of the immune system and epigenetic factors. There are numerous studies that have investigated the mechanisms of MLT on various tumor types, as well as reviews summarizing the results of studies on a number of malignant neoplasms (28–33).

MLT has the capability of scavenging radicals and radical-associated reactants, stimulating the expression of antioxidative enzymes and reducing the expression of pro-oxidants (34,35). The anticarcinogenic actions of MLT associate in part with the antioxidative and free radical scavenging activities. The anti-estrogenic properties of MLT depend on the ability to decrease the expression of estrogen receptor-α (ERα), and to inhibit the binding of the E_2_-ER complex to the estrogen response element on DNA (28,36). These effects are exerted through MLT binding to the specific membrane receptor, MT1. By contrast, the inactivation of calmodulin by MLT is an additional method in which this hormone may interact with the estrogen signaling pathway (37). MLT shares properties with the selective ER and enzyme modulators which explains the oncostatic properties of MLT on estrogen-dependent tumors (28). Other mechanisms of action, including the pro-apoptotic effects of MLT on tumor cells (38) and the inhibition of telomerase activity (39), are only partially understood.

MLT exerts direct antiangiogenic effects through inhibiting vascular endothelial growth factor. Indirect effects are also exhibited by MLT through inhibiting other tumor growth factors, including epidermal growth factor, endothelin-1 and insulin-like growth factor 1, which are significant mitogens that stimulate cancer angiogenesis (40). In addition, MLT neutralizes reactive oxygen species. Studies on the antiangiogenic properties of MLT are of significant importance for possible future clinical applications (28). MLT is also synthesized by lymphoid organs, including bone marrow, the thymus and lymphocytes, and is considered an immunoenhancer agent. The administration of MLT stimulates the production of natural killer cells, monocytes, leukocytes, interleukin (IL)-2, -6 and -12, interferon-γ and TNF-α through binding to specific membrane and nuclear receptors present in these cells (41). Finally, novel roles for MLT in the epigenetic modulation of gene transcription have also been indicated (28).

## 3. Expression and function of MT1 and MT2 receptors

MLT and its metabolites interact with the intracellular protein, calmodulin, RZR/ROR family nuclear-membrane receptors and MT1 and 2 receptors located in the cell membrane (42). The MT1 and 2 receptors were initially referred to as Mel1a and Mel1b, but were later classified as MT1 and MT2 receptors by the International Union of Basic and Clinical Pharmacology (43). The MT1 and 2 receptors are members of the G-protein-coupled receptor (GPCR) family and share a number of their amino acid sequences (44). With the use of recombinant MLT receptors, the MT1 receptor has been shown to be coupled to various G proteins that are able to mediate adenylyl cyclase inhibition and phospholipase Cβ activation. The MT2 receptor is also coupled to the inhibition of adenylyl cyclase and additionally inhibits the soluble guanylyl cyclase pathway (45).

A third member of the MLT receptor family is the X-linked orphan, GPR50 (46), which shares 45% homology with the MT1 and 2 receptors. However, the ligand of GPR50 and its physiological function remain unclear, although an involvement in key hypothalamic functions, including the regulation of the hypothalamopituitary axes, has been indicated (47). Moreover, orphan GPCRs heterodimerize with GPCRs that have identified ligands, resulting in the regulation of the latter GPCR function (48). Deletion of the large C-terminal tail of GPR50 suppresses the inhibitory effect of GPR50 on MT1 without affecting heterodimerization, indicating that this domain regulates the interaction of regulatory proteins to MT1 (49).

Investigation has also been conducted into an MT3 receptor/binding site. Despite MT3 being a presumptive membrane receptor, following stimulation, the transduction cascade and biological consequences have not been elucidated. Moreover, a number of studies support the hypothesis that the MT3 binding site is an enzyme, QR2, rather than a membrane MLT receptor (50). It has been hypothesized that MLT is a co-substrate of QR2, which itself is believed to be a an antioxidant and detoxifying enzyme that changes behavior depending on the co-substrates available. MLT is a naturally occurring substance whose levels consequently fluctuate with the light/dark cycle, the health/disease state and aging. Therefore, these alterations in MLT production, under physiological or pathological conditions, are likely to affect the activity of QR2. However, the hypothesis that MTL is a substrate or co-substrate of this enzyme is controversial (51).

With regard to the mechanisms behind MLT anticancer function in the oral cavity, the present data remain insufficient. Epidermoid carcinoma is one of the most frequent tumors of the oral cavity, with aggressive behavior. In patients with epidermoid carcinoma in whom the presence of the MT1 receptor has been studied through mRNA expression, MT1 has been shown to be diminished or non-existent. This is in contrast to what occurs in the normal epithelium of the oral cavity. It has been shown that in human cancers, including oral squamous cell carcinoma, DNA methylation of 5′-CpG islands (cytosine and guanine separated by phosphate) is a major cause of tumor-suppressor gene inactivity (52). In these tumors, there is an inverse correlation between MT1 receptor expression and DNA methylation. By contrast, the absence of immunoreactive MT1 is associated significantly with a greater tumor size and poorer survival prognosis. In a previous study, restoration of the exogenous MT1 receptor was found to inhibit the growth of epidermoid cells lacking the expression of this receptor (53). In this respect, it has been hypothesized that MTNR1A is a likely target for epigenetic silencing at the homozygously deleted region at 4q35, detected in these tumors (53). In precancerous oral diseases, including leukoplakia and lichen planus, reactive oxygen species are also involved in pathogenesis (54,55). MLT may protect against these pathologies due to its antioxidant properties. However, further studies are required to assess the efficacy of MLT treatment for these cases (56). MLT may be useful to treat diseases of the oral cavity in patients with low concentrations of the hormone, but not where the tissues express MT1 and 2 receptors (57).

## 4. Conclusion

In summary, the role of MLT in carcinogenic processes is being increasingly studied. However, information concerning the involvement of MLT in tumors of the oral cavity is preliminary. The role of MLT, the MT1, 2 and 3 receptors and the RZR nuclear receptors requires further investigation.
